# Medial prefrontal cortical thinning mediates shifts in other-regarding preferences during adolescence

**DOI:** 10.1038/s41598-017-08692-6

**Published:** 2017-08-17

**Authors:** Sunhae Sul, Berna Güroğlu, Eveline A. Crone, Luke J. Chang

**Affiliations:** 1 Department of Psychology, Pusan National University, Busan, Republic of Korea; 2 Developmental and Educational Psychology Unit, Leiden University, Leiden, The Netherlands; 3 Leiden Institute for Brain and Cognition, Leiden University Medical Centre, Leiden, The Netherlands; 4 Department of Psychological and Brain Sciences, Dartmouth College, Hanover, NH USA

## Abstract

Adolescence is a time of significant cortical changes in the ‘social brain’, a set of brain regions involved in sophisticated social inference. However, there is limited evidence linking the structural changes in social brain to development of social behavior. The present study investigated how cortical development of the social brain relates to other-regarding behavior, in the context of fairness concerns. Participants aged between 9 to 23 years old responded to multiple rounds of ultimatum game proposals. The degree to which each participant considers fairness of intention (*i.e*., intention-based reciprocity) vs. outcome (*i.e*., egalitarianism) was quantified using economic utility models. We observed a gradual shift in other-regarding preferences from simple rule-based egalitarianism to complex intention-based reciprocity from early childhood to young adulthood. The preference shift was associated with cortical thinning of the dorsomedial prefrontal cortex and posterior temporal cortex. Meta-analytic reverse-inference analysis showed that these regions were involved in social inference. Importantly, the other-regarding preference shift was statistically mediated by cortical thinning in the dorsomedial prefrontal cortex. Together these findings suggest that development of the ability to perform sophisticated other-regarding social inference is associated with the structural changes of specific social brain regions in late adolescence.

## Introduction

The foundation of modern society is based on large-scale cooperation between genetically unrelated conspecifics^1–4^. Though humans are not the only species to exhibit other-regarding preferences (*e.g*., refs 5–7), the complexity of social interactions has evolved to a much greater extent among humans than other species^3^. This may result from the relatively prolonged cortical development, which continues until late adolescence and young adulthood^8–11^, within regions presumed to comprise the “social brain”, a set of brain regions involved in inferring others’ mental states, such as medial prefrontal cortex (MPFC), posterior cingulate cortex (PCC), temporo-parietal junction (TPJ), and posterior superior temporal sulcus (pSTS)^8, 9, 12, 13^. A critical question, therefore, is how our neural architecture develops to enable the complex computational processing necessary for sophisticated social interactions. We sought to answer this question by investigating the relationship between cortical development of social brain and shifts in other-regarding preferences from childhood to adulthood, in the context of fairness concerns.

Fairness concerns, which play a fundamental role in human cooperation^14^, are based not only on egalitarian preferences for equitable outcomes^15^ (*i.e*., egalitarianism) but also on reciprocity considerations about others’ intentions^16–19^ (*i.e*., intention-based reciprocity). People tend to reciprocate others’ intentions even if the final outcomes are the same^16, 18^. For example, in a standard ultimatum game (UG)^20^, a proposer decides what proportion of an endowment he/she would like to offer to responders and a responder decides whether to accept or reject the proposers’ offer. Even though the money-maximizing strategy is to accept any offer, responders typically *reject* unfair offers smaller than 20% of a total endowment^14^. However, the same unfair offers are more likely to be *accepted* if the proposer demonstrates good intentions by choosing the inequitable division over an even more unfair division in a modified version of the UG, called mini-UG^18^. Interestingly, the relative importance of preferences for egalitarianism and intention-based reciprocity seems to change with age. Several developmental studies have shown that the preference for egalitarianism emerges in early childhood^21–24^ and then shifts towards intention-based reciprocity in adolescence^25–27^. This shift in other-regarding preferences from using simple rule-based strategies (*e.g*., do we have equal shares?) to more sophisticated computations (*e.g*., why did she/he make such decision?) from childhood through adulthood may require cortical maturation of the social brain, because intention-based reciprocity requires a higher-level cognitive ability to infer others’ intention by integrating complex contextual social information. Among the social brain regions, MPFC has been consistently implicated in social inference and mentalizing processes^28–30^. However, there has yet to be a study that links the structural changes in the brain to the behavioral shifts in other-regarding preferences. The present study investigates how cortical development from childhood to adulthood relate to the computational demands associated with different types of other-regarding preferences, namely egalitarianism and intention-based reciprocity.

**Figure 1:**
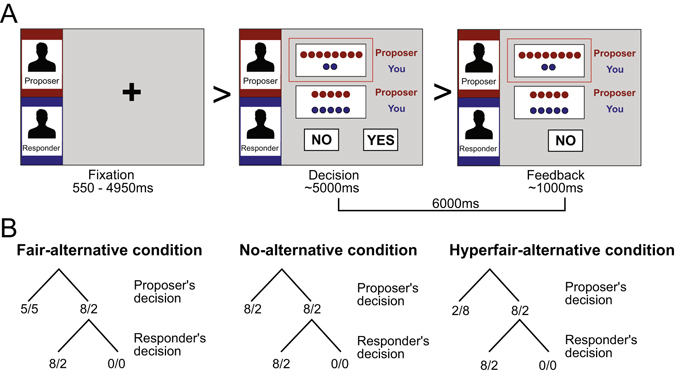
Experimental design. (**A**) An illustration of a single trial of the UG task (fair-alternative condition). Each trial started with the presentation of a fixation crosshair with a jittered inter-stimulus interval (mean = 1530 s, min = 550 ms, max = 4950 ms; optimized with Opt-Seq 2; surfer.nmr.mgh.harvard.edu/optseq;^68^, followed by a decision phase where the participants made decisions after viewing two options available to the proposer and the proposer’s decision. The participants were instructed to make a decision within 5000 ms and their choice was shown on the feedback screen until the end of 6000 ms. (**B**) Examples of decision trees for the fair-alternative, no-alternative, and hyperfair-alternative conditions.

Eighty-eight healthy right-handed participants aged between 9 and 23 years played as a responder in a mini-UG^18, 26^ (see Fig. 1 and Methods). In this task, responders were asked to decide whether to accept or reject a division of money (*e.g*., $8 for the proposer and $2 for the responder) and were given counterfactual information about the alternative split that the proposer could have chosen. The choice sets available to proposers were drawn from three different conditions: fair-alternative (unfair vs fair), hyperfair-alternative (unfair vs hyperfair), and no-alternative (identical unfair). This design has the potential to behaviorally disentangle preferences for egalitarianism and intention-based reciprocity.

**Figure 2:**
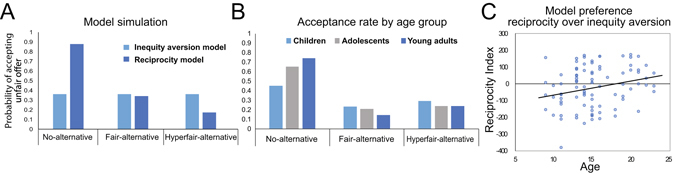
Model simulation and behavioral results. (**A**) Model simulation of the predicted probability of accepting 8/2 offer pitted against different alternative choice sets. (**B**) Average acceptance rates for no-alternative, fair-alternative, and hyperfair-alternative conditions, grouped into children, adolescents, and young adults for illustration purpose. All statistical analyses were performed with age as a continuous variable. (**C**) Model fit difference between the inequity aversion model and reciprocity model (reciprocity index). Relative preference for the reciprocity model increased with age (this result remained significant even after excluding the participant with the lowest reciprocity index on the bottom left of the scatter plot).

We used a computational modeling approach to quantitatively operationalize the type of other-regarding preference employed by each participant^29, 31, 32^. Based on prior work we hypothesized that younger participants would primarily be concerned with minimizing inequity in payoffs and would reject most of the unfair offers^23, 27^. This model of egalitarian other-regarding preference was quantified using the inequity-aversion model^15^. In contrast, we predicted that older participants would utilize a more sophisticated type of reasoning that considers the other players’ intentions^18^. We operationalized this hypothesis using the reciprocity model^17, 19^, which employs psychological game theory to model beliefs directly in the utility function^33^. The inequity-aversion model predicts an equal rejection rate for unfair offers across all experimental conditions, while the reciprocity model predicts that responders will be more likely to accept inequitable offers when he/she believes that the proposer did not have a choice and a decreased acceptance rate when the proposer could have made a more generous offer (see Fig. 2A for model simulation). For each participant, we calculated how well each model explained their data and then identified which regions of the brain mediate the preference shifts.

We derived cortical thickness measures from high resolution structural magnetic resonance imaging (MRI) as a cross-sectional measure of cortical development. Cortical thickness has been reported to be a reliable measure of structural brain development in many studies^8–10, 12, 34^. Based on previous findings that cortical thickness of the social brain linearly decreases with age during adolescence^8–10, 34^, we predicted that cortical thinning of the social brain regions involved in social inference such as the MPFC^4, 28, 29^ would be associated with a shift in other-regarding preferences during adolescence.

## Results

### Behavioral results

#### Average acceptance rate

Figure 2B shows the average acceptance rates for unfair offers by different age groups. Consistent with our prediction that older participants would be more likely to consider the intention of proposers, the decision patterns of older participants matched more closely the acceptance rates predicted by the reciprocity model than the inequity aversion model. Older participants showed higher acceptance rates than younger participants in the no-alternative condition where proposers were unable to make a more equitable offer: age (continuous variable) interacted with the within-subject conditions (no-alternative, fair-alternative, and hyperfair-alternative), with respect to the proportion of accepting unfair offers [*F*(2, 166) = 5.673, *p* = 0.004, *η*_p_^2^ = 0.64]. We did not find any age-related differences in reaction times (all *p*s > 0.05). Note that we divided participants into three age groups only for illustration purpose in Fig. 2B but all the analyses were performed with age as a continuous variable.

#### Computational Modeling

This result was further corroborated by the computational modeling analysis. The inequity aversion model and the reciprocity model were fit to trial-by-trial choices of each individual by maximizing the log-likelihood of the data given the model (see Methods). Each model fit was summarized using the Bayesian Information Criteria (BIC;^35, 36^), which penalizes models that have increased complexity with additional free parameters. We calculated a reciprocity index by subtracting the BIC value for the reciprocity model (RC) from the BIC value for the inequity aversion model (IE) within an individual to capture the extent of favoring the reciprocity model over the inequity aversion model. A multiple regression analysis predicting the reciprocity index by age, controlling for the effects of sex and IQ, showed that the reciprocity model explained the decisions of the older participants better than the inequity aversion model (B = 7.939, SE = 3.465, *t* = 2.291, *p* = 0.024, Fig. 2C).

To better understand this relationship, we performed a simple effects regression analysis on the effect of age on the BIC scores separately for the inequity aversion and reciprocity models. The results showed that the BIC for the inequity aversion model did not change with age (B = 0.633, SE = 1.887, *t* = 0.335, n.s.), while the BIC for the reciprocity model significantly decreased with age (B = − 7.306, SE = 2.987, *t* = −2.446, *p* = 0.016). In other words, older participants were more likely to consider the proposer’s intentions than younger participants when they made choices during the UG task. Unlike the relatively stable preference for egalitarianism, the other-regarding preference for intention-based reciprocity seems to emerge during adolescence, with the gradual shift from egalitarianism to intention-based reciprocity (Fig. 2C).

### Brain structure results

#### Cortical thickness and age

Our primary goal was to investigate the relationship between neuroanatomical development and shifts in other-regarding preferences during adolescence. We hypothesized that the cortical development of the social brain regions, especially the MPFC, would mediate shifts in other-regarding preference. We first examined the effect of age to ensure our data replicated previous findings on the developmental changes in cortical thickness. A vertex-wise general linear model (GLM) with age as a predictor of the cortical thickness, after controlling for the effects of sex and IQ, revealed an overall cortical thinning across the entire brain except for the anterior aspects of temporal lobe (Figure S1A). The correlation between age and cortical thickness was particularly robust in the social brain regions including the MPFC, STS, and TPJ (Figure S1B), consistent with previous findings on the cortical development during adolescence^8, 9^.

#### Cortical thickness and other-regarding preference

**Figure 3:**
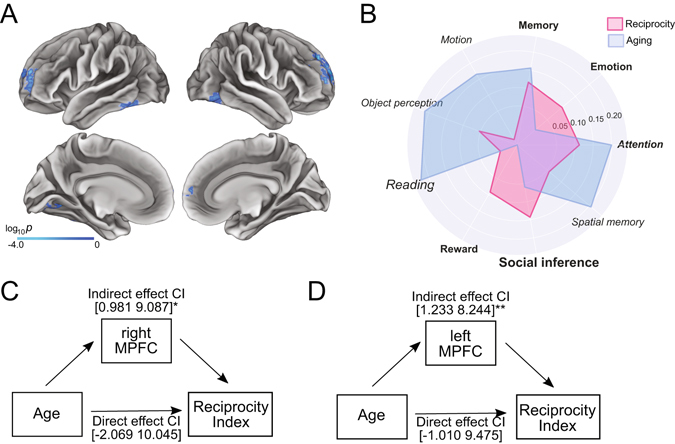
Brain structure results. (**A**) Cortical thickness correlated with reciprocity index. (**B**) Decoding with Neurosynth of the regions associated with reciprocity index (pink) and the regions associated with age (blue). The top 5 topics from the reciprocity map (boldface) and the aging map (italic) are shown in the polar map. The topics that showed the highest correlation in each analysis are marked with larger font. Social inference was the topic that showed the highest correlation with the association between the reciprocity index and the cortical thickness. (**C**) (**D**) Mediation analysis showing the significant indirect effect of age on reciprocity index through the cortical thickness of right and left MPFC. The relationship between age and the fairness consideration were mediated by the developmental changes of cortical thickness of the MPFC, after controlling for the effect of sex and IQ. Solid line and dashed line indicate significant and non-significant pathways, respectively. Indirect effect and direct effect CIs are the 95% confidence intervals of the coefficients obtained from non-parametric bootstrap methods. Brain figure created using Connectome Workbench software (http://www.humanconnectome.org/software/connectome-workbench.html). *p < 0.05. **p < 0.01.

We next identified the regions that are related to preferences for intention-based reciprocity vs. egalitarianism, by performing a vertex-wise GLM analysis with the reciprocity index as a predictor of the cortical thickness, after controlling for the effects of sex and IQ. The thickness of MPFC along with other regions in the posterior temporal lobe implicated in social cognition^12, 37–40^ showed significant correlations with the reciprocity index. Specifically, cortical thickness measures of the bilateral dorsomedial prefrontal cortex (DMPFC; MNI coordinates X = 18, Y = 47, Z = 33; X = −21, Y = 58, Z = 8), bilateral fusiform gyrus (FFG; X = 42, Y = −64, Z = 12; X = −41, Y = −52, Z = −13), and left lingual gyrus (X = −19, Y = −70, Z = −11) were negatively correlated with the reciprocity index (Fig. 3A, Table S5). That is, the model preference for reciprocity over inequity aversion corresponded with decreased cortical thickness. Interestingly, these regions largely overlapped with the age-related regions (Figure S1C), suggesting that the thickness of these regions decrease with age.

#### Meta-analytic decoding with Neurosynth

To better characterize the functions implied by the regions that are associated with the shifts in other-regarding preferences, we conducted meta-analytic decoding using the Neurosynth framework^31, 41–43^ (see Methods for details). This approach enables quantitative reverse inference of the broad possible functions associated with the cortical thickness results. Here we adopted meta-analytic reverse inference maps based on 41 topics from a previous study^41^. Each map contained values that reflect the degree to which each voxel is associated with the corresponding psychological topic. By calculating spatial similarity between each topic-based reverse inference map and the cortical thickness map (*i.e*., correlation between the thickness and the reciprocity index), we identified 16 statistically significant topics that are implied in the regions associated with the development of other-regarding preference (Fig. 3B and Table S6). Interestingly and consistent with our hypothesis, the “social inference” topic was most strongly associated with our cortical thickness map. This finding appeared to be somewhat specific as it was different from the decoding result of the cortical thickness changes associated with growing older (Fig. 3B and Table S7). In addition, we analyzed the terms comprising the social inference topic and found that the most frequent term used in the topic was “infer” (Figure S4). These results suggest that the regions mediating other-regarding preference shift during adolescence are associated with the ability to infer others’ intention.

#### Mediation analysis

As a secondary analysis to examine whether the changes in cortical thickness of the social brain regions could account for the age-related shifts in behavior, we extracted the average cortical thickness measures from each of the five clusters correlated with the reciprocity index in the vertex-wise analysis above (i.e., right FFG, left FFG, and left lingual gyrus; shown in Fig. 3A) and performed a mediation analysis^44, 45^. All five ROIs showed significant indirect effects, such that the developmental change in other-regarding preferences was mediated by the cortical thinning of the bilateral MPFC and the other four ROIs in the posterior temporal lobe (Figs 3C,D, and S2; see Figure S3 for the scatter plots depicting the correlations among age, cortical thickness, and other-regarding preference). Bootstrap analyses^44, 45^ confirmed that the indirect effects through the cortical thickness of all five ROIs were significant (95% confidence interval (CI) for the right MPFC: [0.981 9.087], left MPFC: [1.233 8.244], right FFG: [2.453 10.513], left FFG: [1.814 9.082], left lingual gyrus: [2.360 10.568]; ratio of the indirect to total effect for the right MPFC: 0.503, left MPFC: 0.509, right FFG: 0.690, left FFG: 0.634, left lingual gyrus: 0.758), whereas none of the direct effects were significant (95% CI for the right MFPC: [−2.069 10.045], left MPFC: [−1.010 9.475], right FFG: [−4.496 9.191], left FFG: [−3.282 7.768], left lingual gyrus: [−2.844 7.586]). Even using independently defined ROIs (see Methods), we found a significant indirect effect of the age on the preference shifts through cortical thinning of the DMPFC. Bootstrap analyses^44, 45^ confirmed that the indirect effects through the cortical thickness were significant (95% CI for the right DMPFC: [0.238 6.269], left DMPFC: [0.071 6.830]), while the direct effects from age to the reciprocity index were not significant (95% CI for the right DMFPC: [−1.317 11.977], for the left DMPFC: [−1.008 11.608]).

## Discussion

Successfully navigating the complex social environment of modern society entails inferring others’ intentions, learning who to trust, and developing lasting relationships, alliances, and collaborations^32^. This unique aspect of human society requires developing mental models of others’ beliefs, intentions, and desires, which take considerable effort and computational resources to train. Therefore, it is important to understand how our neural architecture dynamically develops alongside our capacity to engage in more sophisticated types of mental inference during social interactions. The present study sought to answer this question by investigating how cortical development mediates shifts in other-regarding preferences from a simple rule-based egalitarianism to a more complex intention-based reciprocity during adolescence.

A key aspect of our approach is the use of computational models developed in the field of behavioral economics to operationalize and quantify the developmental changes in other-regarding preferences. Specifically, we compared the degree to which participants considered equity of outcomes with the degree to which they considered intentions of their interaction partners, by contrasting two different economic utility models, namely the inequity aversion model^15^ and the reciprocity model^17, 19^. We quantified the relative difference between how well each of these models explained participants’ decisions to correlate preference shifts with cortical thinning across development. Other-regarding preferences appear to change during adolescence from egalitarianism, which focuses on equity of outcomes, to intention-based reciprocity, which considers the inferred intentions of the other player. This finding is well in line with extant evidence that moral reasoning and social decision-making undergo transitions from obeying rigid rules in early childhood to more flexibly considering the thoughts and beliefs of others in later age^22, 23, 27, 46^. Yet, it is intriguing that the preference for egalitarianism over intention-based reciprocity seemed to switch in late adolescence around age 17 and 18 years because the ability to take others’ perspectives, or theory of mind (ToM) is known to develop much earlier^47^. This might reflect the possibility that development of more sophisticated types of other-regarding preferences is not merely based on mentalizing ability, but rely on more complex computations such as differentially weighing outcomes and intentions^27, 48^. For instance, children may stick to an egalitarian principle while adults may consider different types of fairness principles such as egalitarianism *and* reciprocity simultaneously before making a decision. Intriguingly, recent findings on non-social decision-making suggest similar shifts from adopting a simple habitual strategy to utilizing complex evaluative processes during adolescence^49, 50^. Consistent with our findings, such a valuation process is known to be associated with MPFC^28, 51^.

Most importantly, our study highlights a link between changes in other-regarding preferences to structural changes in the brain that occur over adolescence. The shift from egalitarianism to intention-based reciprocity was statistically mediated by cortical thinning in the DMPFC and posterior temporal lobe that are known to be involved in processing social information^4, 8, 11, 12, 28–30, 32, 37–40, 47, 52–56^. The DMPFC in particular has been reliably associated with inferring others’ mental states, commonly referred to as mentalizing or theory of mind processing^8, 37, 38, 47^ and has previously been shown to undergo considerable cortical thinning across adolescence^9^. Although the posterior temporal lobe, which includes the fusiform gyrus, has not been directly associated with high-level social computation in decision making literature, it is interesting to speculate how changes in these regions^11, 56^ might also correlate with developmental changes in other-regarding preferences. This region has been associated with making social inferences in previous studies^53, 54, 57, 58^ and it is possible that our ability to make inferences about others’ mental states tracks with our ability to perceive subtle facial cues^40, 55^.

Another important aspect of our approach is the use of meta-analytic decoding. To assess whether the spatial configuration we observed was specific to social cognition in a more quantitative way, we conducted a whole-brain reverse inference analysis using Neurosynth meta-analytic topics as priors. We found that the social inference topic was the most spatially consistent with our results among 41 topics that covered a broad range of cognitive processes. This result further confirms that the preference shifts from egalitarianism to intention-based reciprocity is likely associated with the development of mental inference ability. Importantly, this finding appeared to be somewhat specific as social inference was not of sufficiently high rank to be statistically significant for the age-related cortical thickness map. Yet, the regions associated with the other-regarding preference shift were also associated with other processes that are related to general cognitive ability such as memory and attention. One potential explanation is that older adolescents have a greater cognitive capacity to perform sophisticated mental reasoning that presumably recruits prefrontal processes and that our effect is not specific to social cognition. To examine this possibility, we measured participants’ IQ as an estimation of general cognitive capacity. We did not find any effect of IQ on our dependent measures and all the effects reported in this article persisted despite statistically controlling for IQ. Moreover, the ability to infer others’ mental states does not necessarily require the exclusive involvement of ‘social’ cognition. It is plausible to expect that development of sophisticated mental models involves general cognitive processes^47, 52^.

In summary, the present study used a novel approach combining economic utility models and cross-sectional cortical thickness measures to demonstrate that structural changes in the social brain during adolescence underlie shifts in other-regarding preferences. Our findings provide an important link between brain and behavior and suggest the importance of structural changes in the MPFC and posterior medial temporal lobe during late adolescence in development of complex mental models which facilitate the ability to infer others’ intentions. These changes in neural architecture may be critical to the emergence of a more sophisticated form of other-regarding preference that might ultimately enable large-scale human cooperation at a societal level.

## Methods

### Participants

Eighty-eight healthy right-handed participants aged between 9 and 23 years (children: N = 17, age = 9–11 years, 6 females; adolescents: N = 46, age = 13–17 years, 23 females; young adults: N = 25, age = 18–23 years, 14 females; Note that we grouped participants for illustration purpose only and age was treated as a continuous variable in all analyses) took part in the study (Table S1 for demographic information). A subset of the current data has been previously reported^26, 59^. Four participants were excluded due to poor image quality, leaving 84 participants included in the cortical thickness analysis. The study protocol was approved by the ethical committee at Leiden University Medical Centre and the experiment was performed in accordance with the relevant guidelines and regulations. All participants provided informed consent and participants younger than 18 years old were accompanied by their parents who also provided consent. Participant’s Intelligence Quotient (IQ) was measured using verbal and nonverbal subtests of Wechsler Intelligence Scales as an estimation of general cognitive capacity. For children, IQ was estimated using the Block design and Similarities subscales of the Wechsler Intelligence Scale for Children (WISC;^60^) and for adolescents and young adults, using the equivalent subscales of the Revised Wechsler Adult Intelligence Scale (WAIS-R;^61^). Adolescents (*M* = 100.96, *SD* = 12.89) had relatively lower IQ than children (*M* = 113.63, *SD* = 9.61; *t*(84) = 2.867, *p* < 0.01) and young adults (*M* = 106.80, *SD* = 12.24; *t*(84) = 0.163, *p* = 0.57). However, IQ scores were not related to any of our primary dependent measures such as the average acceptance rate in the UG task (*r* = −0.13, *p* = 0.21), model fit difference (*r* = 0.11, *p* = 0.31), or cortical thickness (no significant clusters were found after correcting for multiple comparisons). We controlled for the effects of IQ and sex in all statistical analyses reported in the present study.

### Ultimatum game (UG) task

Participants played the role of responder in a modified version of the mini-UG^26^, which was originally described by Falk and colleagues^18^. In this task, the responder (*i.e*., participants) received an online decision ostensibly made by a proposer between two options for sharing a stake with the responder. The proposers’ decisions were predetermined based on a behavioral pilot such that the responder received unfair offers most of the time and fair or hyperfair offers about a third of the trials (see Table S2 for all possible games). Choice sets available to the proposers varied across three conditions. In all conditions, at least one of the two options was an inequitable offer, namely an offer of 2 (8/2) or 3 (7/3). However, the responders were led to believe that the proposers were given different alternative choices to the inequitable offer across the conditions. In the fair-alternative condition, the proposer could choose to offer 5 (5/5). In the hyperfair-alternative condition, the proposer could choose to send 8 (2/8) or 7 (3/7) instead of 2 (8/2) or 3 (7/3). Finally, in the no-alternative condition, the proposer had to choose between the same unfair offers and therefore had no alternative to offer a different amount of money. The three conditions were presented in a pseudorandom order. After the proposer’s choice was presented, the responder had to decide whether to accept or reject the offer. If the offer was rejected, neither player received anything. If the offer was accepted, the two players shared the coins as proposed (Fig. 1). Participants played with coins and were unaware of the conversion rate to Euros. After performing 24 practice trials during which experimenters made sure that all participants understood the task, participants played 168 single-shot games in the scanner with anonymous age and sex matched partners. Participants were told that the proposers’ decisions had been collected in advance in a separate session and the payments for both parties would be implemented based on randomly selected trials. In reality, the offers proposed were generated by computer, based on the data obtained from prior experiments^25^. Payoffs were fixed to five euros for every participant. Experimenters made sure that all participants understood the task during the practice trials. No participant expressed doubt about the cover story in the post-scan interview.

### Decision models

Previous findings^26^ showed that younger children were more likely to focus on the outcome fairness and were less likely to consider the alternative options that reflect the proposers’ intention. In this study, we quantified the degree to which each participant employs these strategies using formal models of utility. This allowed us to track the developmental trajectory of how these strategies are utilized in a more fine-grained fashion. We adopted two models of other-regarding preference from the behavioral economics literature: inequity aversion^15^, which focuses on the outcome fairness (*i.e*., egalitarianism), and reciprocity^17, 19^, which reflects the players’ beliefs about the partners’ intention (*i.e*., intention-based reciprocity). Though the reciprocity model technically had one less free parameter than the egalitarian model, it reflects a psychologically more complex process^17, 19^. While the egalitarian strategy only involves tracking the difference in outcomes between oneself and their partner, the reciprocity strategy requires participants to form a belief about their partner’s kindness given their behavior in each experimental context. Participants then must decide how much kindness to return, which is based on their beliefs about what their partner believes is more kind for a given context. This strategy thus requires second order reasoning, or reasoning about how ones’ actions affect anothers’ beliefs.

In the inequity aversion model, the utility of player *j* (responder)’s choice was defined as follows:

1 $${U}_{j}=\,{x}_{j}-\,\alpha \cdot max({x}_{i}-{x}_{j},\,0)-\beta \cdot max({x}_{j}-{x}_{i},0)$$

where *x*_*i*_ and *x*_*j*_ denote payoffs for player *i* (proposer) and *j*, respectively, *α* and *β* reflect the extent to which disadvantageous inequity (*x*_*i*_ > *x*_*j*_) and advantageous inequity (*x*_*i*_ < *x*_*j*_) influence the utility, and *α* and *β* were constrained between [0,1].

For the reciprocity model, we modified Dufwenberg and Kirchsteiger^17^ and Rabin^19^’s model based on Psychological Game Theory^33^ to match our experimental design. In this model, the utility function of player *j*’s choice was defined as follows:

2 $${U}_{j}={x}_{j}+c\cdot \theta \cdot {k}_{i}\cdot {k}_{j}$$

where *c* = 0 in no-alternative condition, *c* = −1 if *k*_*j*_ *< *0, c = 1 if *k*_*j*_ ≥ 0 in fair- and hyperfair-alternative conditions. *k*_*i*_ and *k*_*j*_ are defined in equations (3) and (4), and *θ* was constrained between [0, 1]. *θ* reflects the extent to which player *j*’s utility is influenced by the alternatives given to player *i*, or in other words, the degree of concern for the intention of player *i* (*i.e*., rec*i*procity).

In equation (3), *k*_*i*_ is player *i*’s kindness to player *j* which is defined by the difference between player *j*’s payoff (*x*_*j*_) from player *i*’s offer and the expected payoff from the full choice set including the alternative given to player *i* (*x*_*j*_’):

3 $${k}_{i}={x}_{j}-\frac{\max ({x}_{j},\,{x}_{j}^{^{\prime} })+\,\min ({x}_{j},{x}_{j}^{^{\prime} })\,}{2}$$

Similarly, *k*_*j*_ represents player *j*’s kindness to player *i*, which is conditional on *k*_*i*_ and can be formalized as:

4 $${k}_{j}={x}_{i}-\frac{\,\max ({x}_{i},{x}_{i}^{^{\prime} })+\,\min ({x}_{j},{x}_{j}^{^{\prime} })}{2}$$

where the payoff of player *i* from player *i*’s offer (*x*_*i*_) equals zero if *k*_*i*_ < 0, assuming that player *j* would not reciprocate. *x*_*i*_ denotes player *i*’s payoff from the alternative given to player *i*.

We used a softmax function to calculate the probability of selecting an action given the utility associated with each choice from *j*’s choices.

5 $${p}_{j}=\frac{{e}^{{u}_{j}}}{{\sum }_{j=1}^{J}\,{e}^{{u}_{j}}}\,$$

We estimated the parameters for each model by maximizing the log likelihood of observing the data given each model, by

6 $$ll=\,\sum _{t=1}^{n}log({p}_{c,j})\,$$

where *c* represents the condition, *j* represents a participant’s decision to accept or reject, *t* represents the trial, and *n* is the total number of trials. We used Matlab (Mathworks, Inc., Natick, MA, USA)’s fmincon optimization function for the parameter search. Software for performing model estimation is freely available at http://cosanlab.com/resources.

After estimating the optimal parameters for each model, we compared the model fits using BIC^35, 36^. In particular, we calculated the reciprocity index by subtracting the BIC value for the reciprocity model from the BIC value for the inequity aversion model within an individual. Because smaller values of BIC indicate a better-fitting model, a reciprocity index larger than zero indicates that the reciprocity model is preferred to the inequity aversion model, and vice versa. In other words, larger reciprocity index reflects an increased likelihood that the responder is employing a reciprocity strategy and considering the proposers’ intentions when making their decision. The reciprocity index was later included in regression analyses as a dependent variable predicted by age and cortical thickness. Additionally, we examined whether individual model parameters (*α* and *β* for the inequity aversion model, and θ for the reciprocity model) were correlated with age and the cortical thickness of the regions correlated with the reciprocity index. The mean parameters and the correlations with age and the MPFC thickness measures are shown in Table S3.

The two models make different predictions about the responder’s decision regarding the alternative choice sets given to proposers. In particular, the inequity aversion model predicts that the probabilities of accepting unfair offers will be the same across the conditions with different alternative choice sets, whereas the reciprocity model predicts the probabilities of accepting unfair offers in the no-alternative condition would be higher than in the fair-alternative and hyperfair-alternative conditions. We performed a model simulation to predict probabilities of accepting 8/2 offer in the no-alternative, fair-alternative, and hyperfair-alternative conditions, using different parameters (*α* and *β* for the inequity aversion model and θ for the reciprocity model were varied between 0 and 1 by an increment of 0.01). Average probabilities of accepting the 8/2 offer predicted in the simulation showed that the reciprocity model reflects the effect of different alternatives from which the participants could infer the proposers’ intention (Fig. 2A).

We set the temperature parameter of the softmax function to 1 for all participants because temperature parameters can absorb stochasticity in participant’s choices, which in turn reduces the extent that model fits can capture individual variability^62^. When we examined the models including the temperature parameter, the inverse temperatures of the reciprocity model were positively (but not significantly) correlated with age and negatively correlated with the MPFC thickness measures, resembling the BIC of our original model. These results are reported in Table S4.

### MRI data acquisition and cortical thickness analysis

Brain images were acquired on a 3 T MRI scanner (Achieva, Philips Medical Systems, Best, The Netherlands) at the university medical center. T2*-weighted functional images were collected while participants performed the UG task, a subset of which were reported in previous studies^26, 59^. High-resolution T1-weighted anatomical scans (TR = 9.778 ms; TE = 4.600 ms; flip angle = 8°; FOV = 240mm, 256 × 256 matrix; 0.875 × 0.875 × 1.200 mm in-plane resolution) were obtained after the functional scans. In the present study, we only report the structural data to stay focused on the relationship between structural shifts and behavioral development. We believe that including functional data is beyond the scope of the present study and hope that further research directly linking structural changes to functional changes could reveal a more complete picture of social brain development.

Cortical thickness was estimated from the T1 images, using FreeSurfer v.5.1.0. software (freely available for download: http://surfer.nmr.mgh.harvard.edu/). Technical details and validation of the automated procedures are described in prior publications (*e.g*., refs 63–65). In brief, we followed the automated surface-based pipeline, which consisted of motion correction, affine registration of the T1 images with MNI305 atlas via Talairach transformation, intensity normalization, removal of non-brain tissue, segmentation, and surface deformation including inflation, spherical registration, and cortical parcellation. The initial surface generated from these procedures was then refined to follow intensity gradients between the white and gray matter (*i.e*., white surface) and the gray matter and cerebral spinal fluid (*i.e*., pial surface). The cortical thickness was estimated from the distance between the pial and white surfaces at each vertex. For the group-level statistical analyses, the cortical thickness maps were smoothed with a 15mm full-width of half maximum Gaussian kernel. Whole-brain vertex-wise analyses were performed using general linear model (GLM) to identify the regions correlated with age and reciprocity index, controlling for sex and IQ. Note that in the GLM average cortical thickness was controlled for through the intercept. Monte-Carlo simulations with a cluster level p-value of 0.005 were performed for multiple comparisons correction^66^. The whole-brain results were visualized using Connectome Workbench software (http://www.humanconnectome.org/software/connectome-workbench.html).

### Meta-analytic decoding with Neurosynth

To understand the functional implications of our cortical thickness results we used meta-analytic reverse inference using the Neurosynth framework (http://neurosynth.org;^43^). In brief, we adopted topic-based maps from a previous study^41^, which are based on 80 topics generated using Latent Dirichlet Allocation topic modeling of the abstracts of 9,204 functional neuroimaging (fMRI) articles where terms are nested within documents. Topics with low loadings (n = 7) or containing non-psychological content (*e.g*., methodological contents, n = 25) or clinical content (e.g., depression, n = 7) were excluded. For the remaining 41 topics, meta-analytic reverse inference maps were generated based on the association between each topic and activation at each voxel reported in the Neurosynth dataset (for details of the topic modeling procedure, see^41, 42^. The meta-analytic reverse inference maps contained the values that quantify the degree to which activation at each voxel was associated with the corresponding topic. For decoding, the cortical thickness surface map was converted into MNI space to match the Neurosynth reverse inference map. We then calculated spatial correlations using a Spearman ranked correlation between the meta-analytic reverse inference map for each topic and the cortical thickness map (*i.e*., the map with −log_10_ *P* that reflects the strength of correlation between the thickness and reciprocity index) for voxels that were at the intersection of the two maps (see^31, 41^). For each topic we calculated the empirical likelihood of obtaining the correlation values using a permutation test with 10,000 samples. The topics were ranked according to the resulting correlation coefficients and we calculated the empirical likelihood that a given correlation ended up at particular rank by permuting the 41 correlations 10,000 times and calculating number of instances that a given correlation exceeded the mean correlation of all lower ranks. For both analyses, we provide a correction for the number of multiple comparisons using the False Discovery Rate with q < 0.05. To examine whether these topics are specific to the preference shifts, we ran the same analysis on the regions associated with age-related changes in cortical thickness. The top five topics from each map were selected to generate a polar map visualizing the relative strength of association between each topic and the cortical thickness results (Fig. 3B), but all results can be viewed in Tables S6 and S7. Note that we sorted the topics from the smallest (negative) to the largest (positive) because cortical thickness was negatively correlated with our variable of interest (*i.e*., reciprocity index).

### Mediation analyses

We examined the relationship between the cortical development and other-regarding preference shift by testing the indirect effect of age on reciprocity index through cortical thickness^44, 45^. We defined the clusters significantly correlated with reciprocity index as regions of interest (*i.e*., the regions shown in Fig. 3A: bilateral MPFC, bilateral FFG, and left lingual gyrus). The mediation model included the age as an independent variable, reciprocity index as a dependent variable, and the average thickness of each ROI as a mediator, while controlling for the effect of sex and IQ (Fig. 3C,D, and Figure S2). The significance of the indirect effect was tested using bias-corrected bootstrap 95% confidence interval. We attempted to minimize circularity in this analysis by only examining brain results that were significantly associated with the reciprocity index (path B), which is not related to the assumptions necessary for the mediation effect: the regions correlated with age or the conjunction between the effect of age and reciprocity index. For completeness, we also performed the same mediation analysis with independently defined DMPFC ROIs based on Desikan-Killiany Atlas^67^ provided by FreeSurfer v.5.1.0. software (http://surfer.nmr.mgh.harvard.edu/). In particular, we extracted the average thickness measures of the superior frontal cortex and rostral middle frontal cortex in the left and right hemispheres. Our results do not appear to be dependent on the specific voxels that were significant from our primary analysis, however, as the results remained the same using the independently defined DMPFC ROI^67^. We note that the possibility of other brain regions that might additionally mediate this relationship, which could be uncovered in future work using a whole-brain approach.

## Electronic supplementary material

Supplemental Information
