# Supplementary material for: Medial prefrontal cortical thinning mediates shifts in other-regarding preferences during adolescence
Source: Sci Rep. 2017 Aug 17;7:8510. doi: 10.1038/s41598-017-08692-6 (PMC5561198; doi:10.1038/s41598-017-08692-6)
Supplement: Supplementary file 1 — Supplemental Information [file 41598_2017_8692_MOESM1_ESM.pdf]

Supplemental Information
for
Medial prefrontal cortical thinning mediates shifts in other-regarding preferences during adolescence

Sunhae Sul[1], Berna Güroğlu[2,3], Eveline A. Crone[2,3], Luke J. Chang[4*]

[1]Department of Psychology
Pusan National University
Busan, Republic of Korea

[2]Developmental and Educational Psychology Unit
Leiden University
Leiden, The Netherlands

[3] Leiden Institute for Brain and Cognition
Leiden University Medical Centre
Leiden, The Netherlands

[4]Department of Psychological and Brain Sciences
Dartmouth College
Hanover, NH

Figure S1. Changes in cortical thickness associated with age. (A) Cortical thickness maps correlated with age with uncorrected statistical threshold of $p < 0.005$. (B) Cortical thickness maps correlated with age with significant clusters after correcting for multiple comparison using Monte-Carlo simulation. (C) Overlap between the regions correlated with age and the reciprocity index. Brain figure created using Connectome Workbench software (http://www.humanconnectome.org/software/connectome-workbench.html).

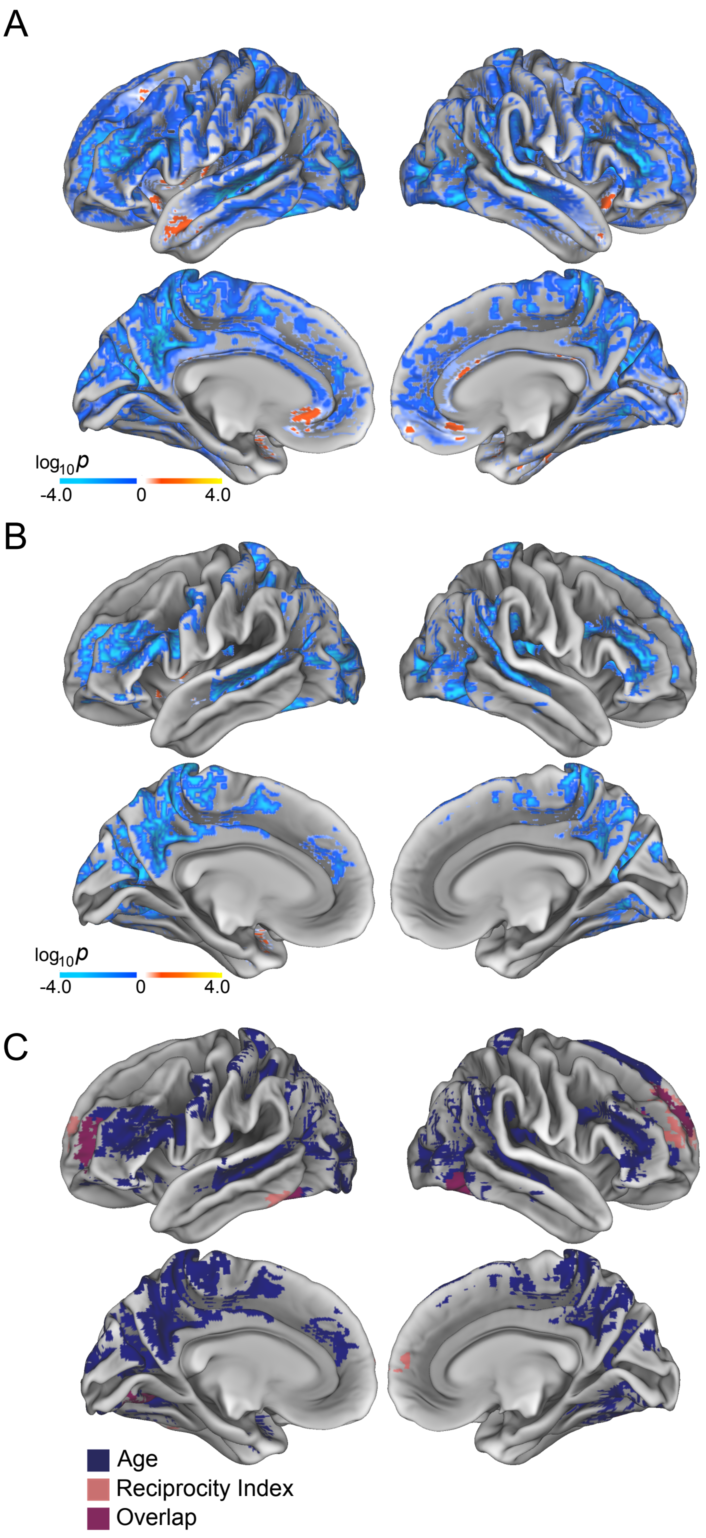

Figure S2. Fusiform gyrus and left lingual gyrus mediates relationship between age and Reciprocity model fit. Numbers next to the arrows indicate standardized beta coefficients, after controlling for the effect of sex and IQ. Indirect effect and direct effect CIs are the 95% confidence interval of the coefficients obtained from non-parametric bootstrap methods. ** $p < 0.01$.

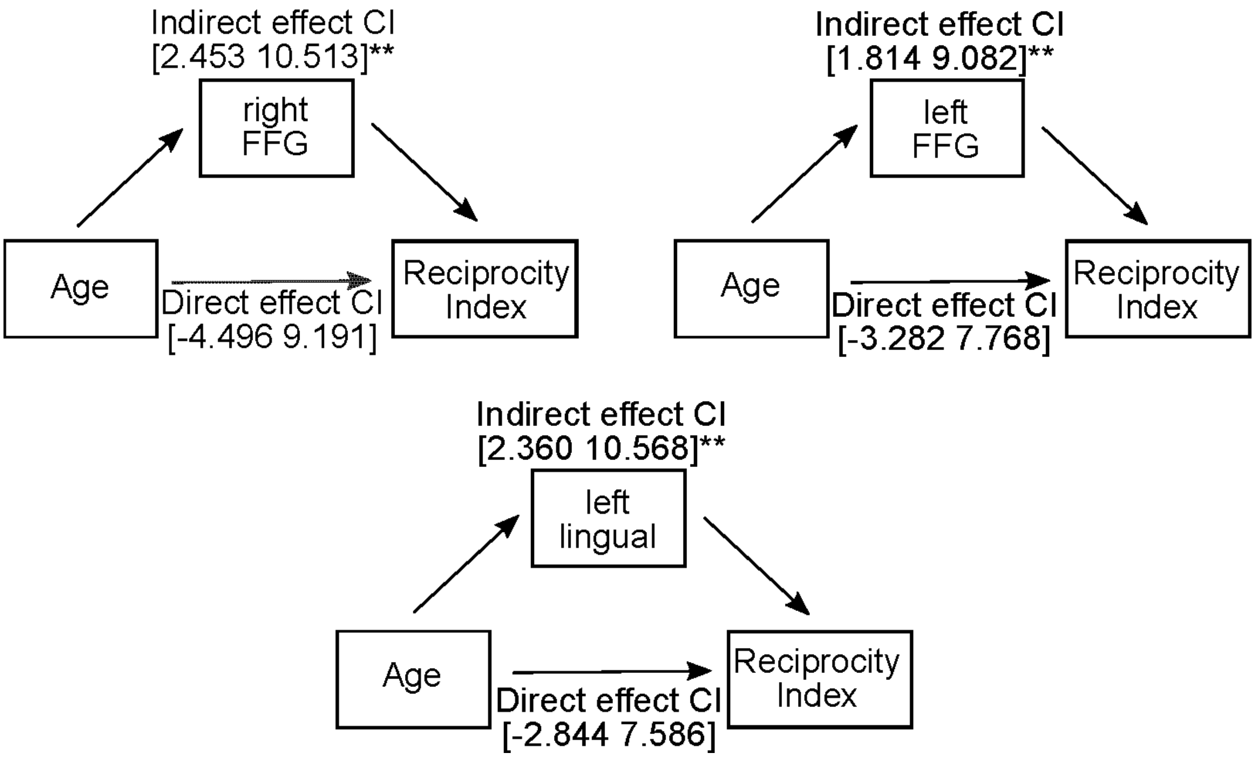

Figure S3. Scatter plots showing the relationships among age, thickness of the five ROIs, and reciprocity index.

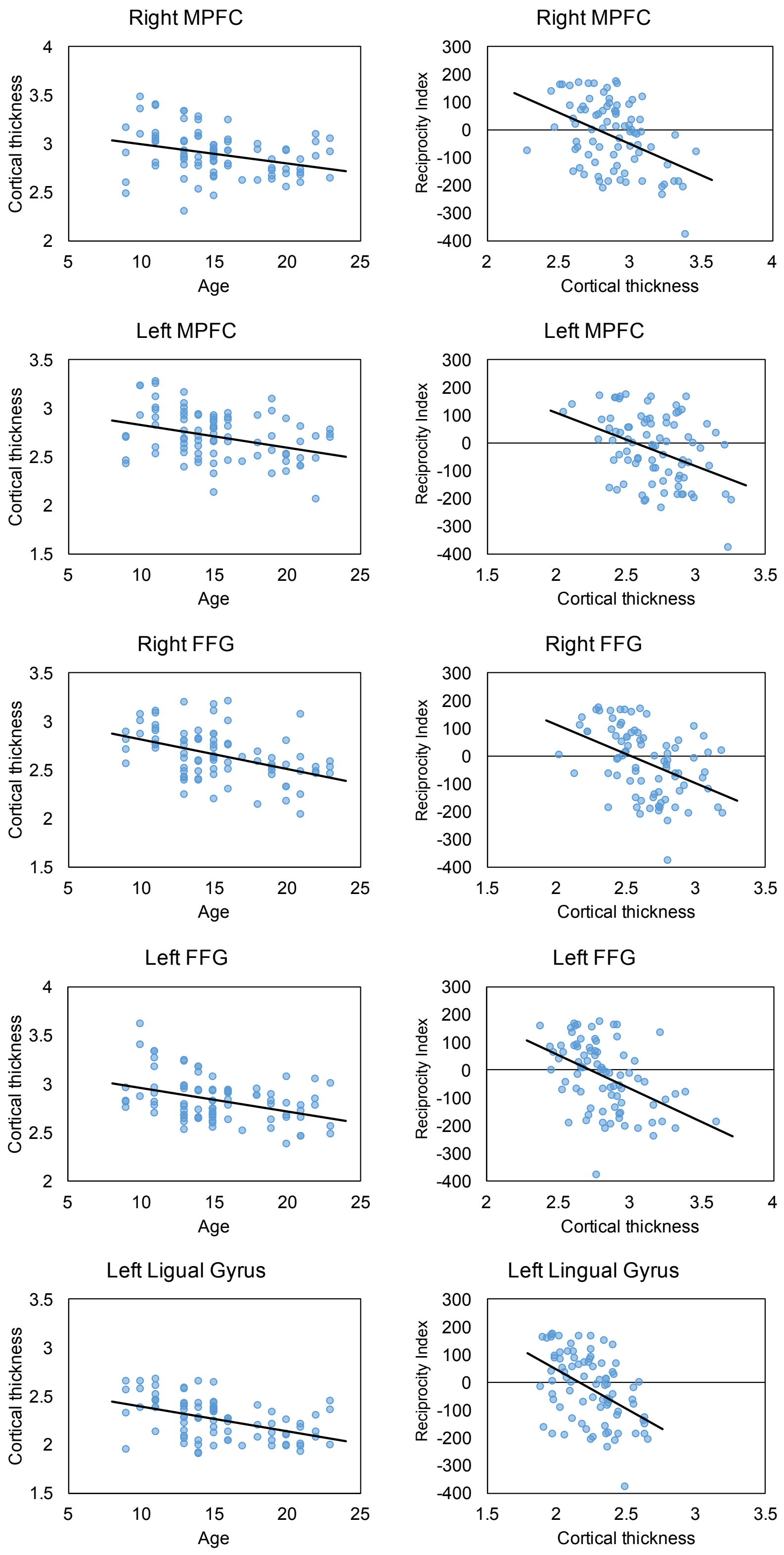

Figure S4. Word cloud of terms comprising social inference topic. Size and transparency of the words reflect the frequency.

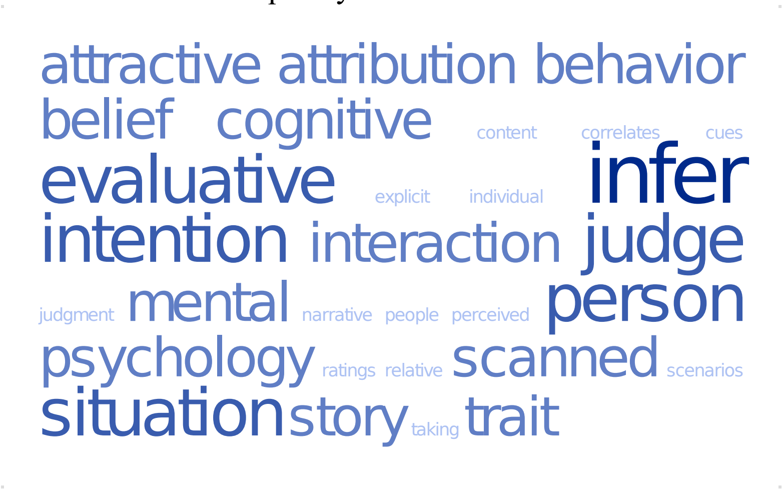

Tables

Table S1. Demographic information of the participants

Age group	Mean age (SD)	Age range	Number of participants	Male	Female	Mean IQ (SD)
Children	10.35 (0.86)	9-11 yrs	17	11	6	113.62 (9.60)
Adolescents	14.39 (1.08)	13-17 yrs	46	23	23	100.96 (12.89)
Young adults	20.24 (1.69)	18-23 yrs	25	11	14	106.8 (12.24)

Table S2. Experimental conditions and mean acceptance rate

Condition	Options	Offer	Number of trials	Mean acceptance rate (SD)
No-alternative	7:3 vs. 7:3	7:3	21	0.73 (0.38)
	8:2 vs. 8:2	8:2	21	0.54 (0.41)
Fair-alternative	7:3 vs. 5:5	7:3	21	0.31 (0.37)
		5:5	9	0.97 (0.12)
	8:2 vs. 5:5	8:2	21	0.08 (0.18)
		5:5	12	0.97 (0.09)
Hyperfair-alternative	7:3 vs. 3:7	7:3	21	0.37 (0.37)
		3:7	9	0.96 (0.10)
	8:2 vs. 2:8	8:2	21	0.13 (0.24)
		2:8	12	0.96 (0.10)

Table S3. Model parameters estimated from the inequity aversion and reciprocity models

Model	Parameters	Mean	Correlation with age (r)	Correlation with right MPFC thickness (r)	Correlation with left MPFC thickness (r)
Inequity Aversion	Alpha	0.63	-0.09	0.12	0.19
	Beta	0.24	-0.01	0.06	0.07
Reciprocity	Theta	0.67	0.08	-0.14	-0.07

Table S4. Model parameters estimated from the inequity aversion and reciprocity models with stochastic choices

Model	Parameters	Mean	Correlation with age (r)	Correlation with right MPFC thickness (r)	Correlation with left MPFC thickness (r)
Inequity Aversion	Alpha	0.68	-0.06	0.06	0.09
	Beta	0.14	-0.05	0.07	0.17
	Phi[1]	0.73	-0.03	0.21	0.18
Reciprocity	Theta	0.74	0.00	-0.07	-0.03
	Phi [1]	0.57	0.21	-0.32**	-0.31**

[1] inverse temperature parameter of softmax function.

** $p < 0.01$.

Table S5. Brain regions negatively correlated with reciprocity index

	Cluster size (mm^2)	MNI coordinates			cluster-wise p-value
		X	Y	Z	
R MPFC	1515.73	18	47	33	0.0001
R Fusiform Gyrus	680.25	42	-64	-12	0.0365
L Lingual Gyrus	949.92	-19	-70	-11	0.0046
L Fusiform Gyrus	733.07	-41	-52	-13	0.0205
L MPFC	1072.24	-21	58	8	0.0013

Table S6. Neurosynth meta-analytic decoding of reciprocity cortical thickness map

Rank	Topic	Topic Correlation	Topic Correlation p-value	Topic Rank p-value
1	Social inference	-0.14	0*	0*
2	Memory	-0.12	0*	0*
3	Attention	-0.11	0*	0*
4	Emotion	-0.10	0*	0*
5	Reward	-0.09	0*	0*
6	Recognition	-0.09	0*	0*
7	Evaluation	-0.07	0*	0*
8	Anxiety	-0.06	0*	0*
9	Spatial memory	-0.06	0*	0*
10	Object perception	-0.05	0*	0*
11	Episodic	-0.05	0*	0*
12	Priming	-0.05	0*	0*
13	Social	-0.05	0*	0*
14	Color	-0.03	0*	0*
15	Anticipation	-0.03	0.001*	0.002*
16	Eating	-0.03	0*	0.004*
17	Semantic	-0.02	0.002*	0.02
18	Cognitive control	-0.02	0.013*	0.053
19	Executive control	-0.02	0.014*	0.065
20	Fear	-0.02	0.024*	0.097
21	Conflict	-0.01	0.029	0.119
22	Error monitoring	-0.01	0.048	0.156
23	Mental imagery	-0.01	0.19	0.299
24	Categorical perception	-0.01	0.234	0.337
25	Reading	0.01	0.177	0.666
26	Abstract reasoning	0.01	0.055	0.76
27	Context	0.02	0.017*	0.82
28	Language	0.03	0*	0.933
29	Autonomic	0.03	0*	0.931
30	Learning	0.03	0*	0.934
31	Motion	0.04	0*	0.951
32	Eye movement	0.05	0*	0.986
33	Action	0.08	0*	0.998
34	Language lateralization	0.09	0*	0.999
35	Polymodal sensory	0.09	0*	0.998
36	Pain	0.09	0*	0.995
37	Target detection	0.12	0*	0.996
38	Auditory	0.12	0*	0.978
39	Motor	0.12	0*	0.927
40	Somatosensory	0.16	0*	1
41	Music	0.16	0*	1

* significant using FDR $q < 0.05$ correction.

Table S7. Neurosynth meta-analytic decoding of age cortical thickness map

Rank	Topic	Topic Correlation	Topic Correlation p-value	Topic Rank p-value
1	Reading	-0.222645133	0*	0*
2	Object perception	-0.211806432	0*	0*
3	Spatial memory	-0.205708694	0*	0*
4	Attention	-0.200313145	0*	0*
5	Motion	-0.166167188	0*	0*
6	Mental imagery	-0.157225463	0*	0*
7	Memory	-0.156450231	0*	0*
8	Executive control	-0.138897451	0*	0*
9	Categorical perception	-0.136990315	0*	0*
10	Color	-0.130740671	0*	0*
11	Priming	-0.112213312	0*	0*
12	Learning	-0.106640736	0*	0*
13	Recognition	-0.096279371	0*	0*
14	Semantic	-0.088887417	0*	0*
15	Action	-0.080928503	0*	0.0004*
16	Cognitive control	-0.076682518	0*	0.0036*
17	Language	-0.074247079	0*	0.0071*
18	Polymodal sensory	-0.072768221	0*	0.0169*
19	Eye movement	-0.069071945	0*	0.0343
20	Anticipation	-0.060874163	0*	0.1084
21	Social inference	-0.059903825	0*	0.1288
22	Language lateralization	-0.057306179	0*	0.1685
23	Abstract reasoning	-0.031124545	0*	0.6511
24	Conflict	-0.019294962	0.0069*	0.8145
25	Motor	-0.01759082	0.0112*	0.8244
26	Emotion	-0.008414429	0.14	0.8944
27	Social	0.008048269	0.1525	0.9657
28	Auditory	0.011871857	0.0653	0.9737
29	Target detection	0.014128674	0.0359	0.9679
30	Context	0.027444765	0.0004*	0.9838
31	Error monitoring	0.028340559	0.0002*	0.9824
32	Reward	0.056387352	0*	0.9961
33	Somatosensory	0.058341068	0*	0.9946
34	Fear	0.065735298	0*	0.9944
35	Episodic	0.074329039	0*	0.9918
36	Music	0.07632704	0*	0.9848
37	Eating	0.091785663	0*	0.9802
38	Anxiety	0.108635113	0*	0.9683
39	Evaluation	0.121061793	0*	0.9268
40	Autonomic	0.20486075	0*	1
41	Pain	0.223064454	0*	1

* significant using FDR $q < 0.05$ correction.
